# Development and Validation of a Sepsis Mortality Risk Score for Sepsis-3 Patients in Intensive Care Unit

**DOI:** 10.3389/fmed.2020.609769

**Published:** 2021-01-21

**Authors:** Kai Zhang, Shufang Zhang, Wei Cui, Yucai Hong, Gensheng Zhang, Zhongheng Zhang

**Affiliations:** ^1^Department of Critical Care Medicine, Second Affiliated Hospital, Zhejiang University School of Medicine, Hangzhou, China; ^2^Department of Cardiology, Second Affiliated Hospital, Zhejiang University School of Medicine, Hangzhou, China; ^3^Department of Emergency Medicine, Sir Run-Run Shaw Hospital, Zhejiang University School of Medicine, Hangzhou, China

**Keywords:** sepsis-3.0, critical care, intensive care unit (ICU), machine learning, mortality prediction model, severity score system

## Abstract

**Background:** Many severity scores are widely used for clinical outcome prediction for critically ill patients in the intensive care unit (ICU). However, for patients identified by sepsis-3 criteria, none of these have been developed. This study aimed to develop and validate a risk stratification score for mortality prediction in sepsis-3 patients.

**Methods:** In this retrospective cohort study, we employed the Medical Information Mart for Intensive Care III (MIMIC III) database for model development and the eICU database for external validation. We identified septic patients by sepsis-3 criteria on day 1 of ICU entry. The Least Absolute Shrinkage and Selection Operator (LASSO) technique was performed to select predictive variables. We also developed a sepsis mortality prediction model and associated risk stratification score. We then compared model discrimination and calibration with other traditional severity scores.

**Results:** For model development, we enrolled a total of 5,443 patients fulfilling the sepsis-3 criteria. The 30-day mortality was 16.7%. With 5,658 septic patients in the validation set, there were 1,135 deaths (mortality 20.1%). The score had good discrimination in development and validation sets (area under curve: 0.789 and 0.765). In the validation set, the calibration slope was 0.862, and the Brier value was 0.140. In the development dataset, the score divided patients according to mortality risk of low (3.2%), moderate (12.4%), high (30.7%), and very high (68.1%). The corresponding mortality in the validation dataset was 2.8, 10.5, 21.1, and 51.2%. As shown by the decision curve analysis, the score always had a positive net benefit.

**Conclusion:** We observed moderate discrimination and calibration for the score termed Sepsis Mortality Risk Score (SMRS), allowing stratification of patients according to mortality risk. However, we still require further modification and external validation.

## Introduction

Being a life-threatening organ dysfunction due to a dysregulated host response to infection, sepsis is considered a major global health problem (1, 2). According to the latest Global Burden of Diseases study, ~48.9 million sepsis cases were reported worldwide in 2017 despite the decline in incidence and mortality. A total of 11.0 million patients died from sepsis and its complications, which accounted for 19.7% of deaths worldwide (3). In the intensive care unit (ICU), sepsis remains a significant cause of morbidity and mortality. According to the ICON study, 29.5% of the patients suffered from sepsis during their ICU stay. The ICU mortality rate was significantly higher in septic patients (25.8%) than the whole population (16.2%) (4). Since rapid treatment could improve the outcomes in septic patients, early identification, and risk assessment are of vital importance (5, 6). A pragmatic scoring system could help clinicians make decisions by identifying high-risk patients and providing the probability of death.

To characterize disease severity and predict its outcome, various severity scores have been widely used in the ICU (7). However, in septic patients, the clinical application remains limited because sepsis's pathogenesis is complicated, and no single score has been developed. For example, the Acute Physiology and Chronic Health Evaluation II (APACHE II) score underestimated the risk of death for septic patients in the ICU (8). Similarly, the Simplified Acute Physiology Score II (SAPS II) showed poor calibration in external validation studies (9, 10). Besides the traditional ICU scoring systems, sepsis mortality prediction models based on machine learning algorithms have been published by some researchers. These models, derived from big medical datasets, could accurately predict mortality with good discrimination for septic patients (11–14). However, most of the models were designed for patients with severe sepsis or septic shock, and none of these were developed from the sepsis-3 patient population. Johnson et al. compared five different methods for screening patients with sepsis, and showed that sepsis-3 criteria provided temporal context, possessed high construct validity and were less influenced by coding changes (15). Therefore, screening patients with sepsis by using the sepsis-3 criteria was considered an optimal method in the electronic database.

Based on sepsis-3 criteria and the Medical Information Mart for Intensive Care III (MIMIC III) database, we aimed to develop a Sepsis Mortality Risk Score (SMRS) by Least Absolute Shrinkage and Selection Operator (LASSO) technique, assess its predictive ability, and compare it with traditional severity scores in the validation dataset from the eICU Collaborative Research Database (eICU). In addition, we built four machine learning models to predict 30-day mortality for sepsis-3 patients.

## Materials and Methods

### Data Source and Participants

We extracted data from the MIMIC III (16) and eICU database (17), respectively. We included adult patients admitted to the ICU with sepsis. Sepsis was identified based on sepsis-3 criteria, which included suspected infection and a Sequential Organ Failure Assessment (SOFA) score ≥ 2 (1). For sepsis patient selection, a previous study was referred for identifying the sepsis-3 cohort from MIMIC III (15). We excluded the following patients: (1) non-adults (<16 years old), (2) multiple admissions, (3) receiving cardiothoracic surgical service (their postoperative physiologic derangements or not translating to the same mortality risk as others), (4) with metastatic cancer (inflammatory and immune response different from others); (5) with suspected infection more than 24 h before or after ICU admission (patients admitted to ICU with sepsis), and (6) missing important data (demographics, variables for calculating traditional severity scores).

### Data Extraction

From the MIMIC III and eICU database, we extracted the following information: (1) demographic information; (2) ICU details including vital sign data, laboratory data, respiratory support, renal replacement therapy; and (3) traditional severity scores including SAPS II, Acute Physiological Score III (APS III), Logistic Organ Dysfunction System (LODS), Oxford Acute Severity of Illness Score (OASIS), SOFA, System Inflammatory Reaction Syndrome (SIRS), and quick SOFA (qSOFA). During the first 24 h of ICU admission, all variables were recorded.

### Outcome and Sample Size

Patients who died within 30 days inside or outside the hospital were considered as primary outcome events. We based our sample size calculation on the primary outcome. The sample size was defined as having at least 10 outcome events per variable (EPV) per estimated parameter according to a previous study (18). Our sample and the number of events exceeded that determined by the EPV approach.

### Missing Data

For the development dataset from the MIMIC III database, we handled variables with missing values <20% by a mean value imputation method. Since serum lactate was considered an important predictor, if lactate data on day 1 was missing, the available data on day 2 or day 3 was used. If there was no lactate value in the first 3 days, we used regression imputation to handle the missing data. To calculate severity scores in the eICU database, patients with missing parameters were excluded from this analysis.

### Statistical Analysis

Continuous variables were reported as median and interquartile range, and two groups were compared by the Mann–Whitney *U*-test. Categorical variables were reported as the number and proportion and were compared with the Chi-square test. The variance inflation factor (VIF) was calculated to verify whether multicollinearity existed in the regression model.

In the development set, we used the LASSO method to select the most useful predictive variables (19). We plotted the continuous variables against 30-day mortality and determined the cutoff value based on the Loess smoothing function and the Youden index (20). Continuous variables were made into dichotomous or dummy variables by the cutoff points. Final variables were entered into a logistic regression, and for each risk predictor, the odds ratio was rounded into an integer value to generate the SMRS. The final score was classified into four risk groups: low (<5%), moderate (5–20%), high (20–50%), and very high (>50%). The survival curves of each mortality risk group were depicted by the Kaplan–Meier method and compared by the log-rank test.

The SMRS was validated in the validation set. To assess discrimination, the Area Under the Curve (AUC) for SMRS and other severity scores was calculated. Calibration was assessed by the calibration slope and the Brier value. To determine the clinical usefulness of the SMRS by quantifying the net benefit at different threshold probabilities, we conducted the decision curve analysis (DCA) (21).

Moreover, the discrimination of four machine learning algorithms in predicting mortality for sepsis-3 patients was compared. In the development set, we developed the logistic regression model, the multivariate adaptive regression splines (MARS) model, the random forest model, and the eXtreme Gradient Boosting (XGBoost) model. The discrimination was validated externally by AUC in the eICU database.

We performed all statistical analyzes using software version 3.6.0 (R Foundation for Statistical Computing).

## Results

### Participants

Our study was reported according to the guidelines of the TRIPOD statement (Checklist in Additional File 1) (22). The initial research identified 23,620 ICU admissions from the MIMIC III database. A total of 5,443 adult patients meeting the sepsis-3 criteria were analyzed, including 907 non-survivors and 4,536 survivors. The baseline characteristics of all patients, survivors, and non-survivors are described in Table 1. While data extraction, we excluded body mass index, albumin, bands, and bilirubin from the analysis because of the large portion of the missing value (>20%). For other variables, the missing value was <10% (Additional File 2). We assigned 5,658 septic patients (1,042 deaths, mortality rate 20.1%) from the eICU database with complete data to the validation set. Comparisons of basic characteristics between development and validation sets are recorded in Additional File 3.

**Table 1:** Baseline characteristics of participants in development set.

**Variables**	**All (*n* = 5,443)**	**Survivors (*n* = 4,536)**	**Non-survivors (*n* = 907)**	***P*-value**
**Age, years**	67.0 (54.0–80.0)	66.0 (53.0–78.0)	75.0 (61.0–84.0)	<0.001
**Gender**, ***n***				0.182
Male	3,020 (55.5)	2,535 (55.9)	485 (53.5)	
Female	2,423 (44.5)	2,001 (44.1)	422 (46.5)	
**Ethnicity**, ***n***				<0.001
White	3,945 (72.5)	3,309 (72.9)	636 (70.1)	
Black	475 (8.7)	421 (9.3)	54 (6.0)	
Others	1,023 (18.8)	806 (17.8)	217 (23.9)	
**Admission type**, ***n***				<0.001
Emergency	5,061 (93.0)	4,175 (92.0)	886 (97.7)	
Others	382 (7.0)	361 (8.0)	21 (2.3)	
**Comorbidities**, ***n***				
Heart failure	957 (17.6)	742 (16.4)	215 (23.7)	<0.001
Hypertension	868 (15.9)	701 (15.5)	167 (18.4)	0.026
COPD	1,103 (20.3)	889 (19.6)	214 (23.6)	0.006
Diabetes	1,563 (28.7)	1,298 (28.6)	265 (29.2)	0.715
Renal failure	1,000 (18.4)	799 (17.6)	201 (22.2)	0.001
Hepatopathy	544 (10.0)	429 (9.5)	115 (12.7)	0.003
Lymphoma	95 (1.7)	74 (1.6)	21 (2.3)	0.151
**Need RRT**, ***n***	395 (7.3)	281 (6.2)	114 (12.6)	<0.001
**Need mechanical ventilation**, ***n***	2,638 (48.5)	2,080 (45.9)	558 (61.5)	<0.001
**Severity score**				
SAPS II	39 (31–50)	37 (29–46)	53 (42–65)	<0.001
APS III	48 (36–63)	45 (34–57)	67 (51–87)	<0.001
OASIS	35 (29–41)	34 (28–39)	42 (36–49)	<0.001
LODS	5 (3–7)	4 (3–6)	7 (5–10)	<0.001
SOFA	5 (3–7)	5 (3–7)	7 (5–11)	<0.001
SIRS	3 (2–4)	3 (2–4)	3 (3–4)	<0.001
qSOFA	2 (2–2)	2 (2–2)	2 (2–3)	<0.001

*Data are expressed as frequencies (percentage) or median (interquartile range). The results of the comparison between the two groups was analyzed by Mann–Whitney test for continuous variables or the chi-squared test for categorical variables*.

*RRT, Renal Replacement Therapies; COPD, Chronic Obstructive Pulmonary Disease; SAPS II, Simplified Acute Physiological Score II; APS III, Acute Physiological Score III; OASIS, Oxford Acute Severity of Illness Score; SIRS, Systemic Inflammatory Response Syndrome; qSOFA, quick Sequential Organ Failure Assessment; LODS, Logistic Organ Dysfunction System; SOFA, Sequential Organ Failure Assessment*.

### Model Development

**Figure 1:**
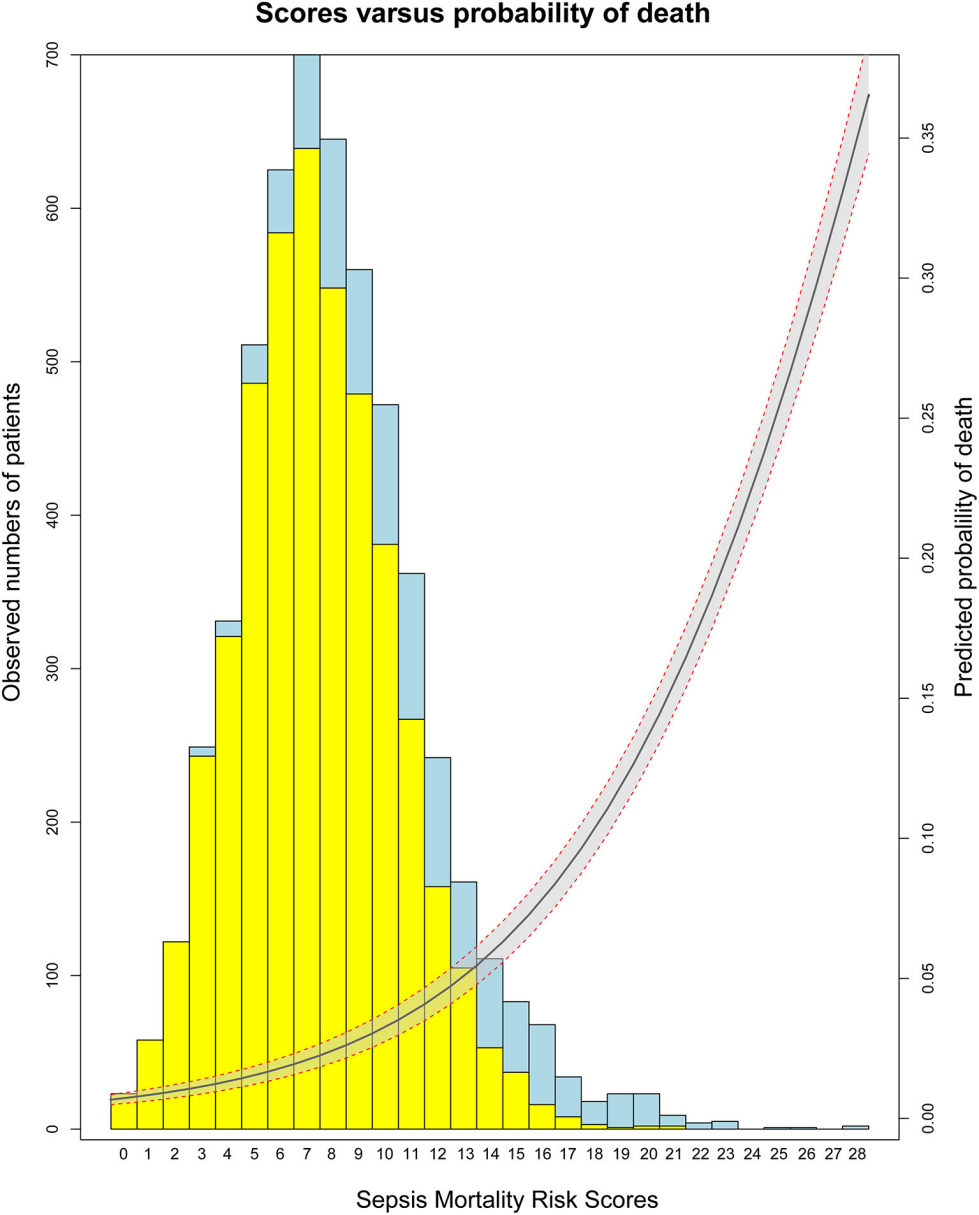
The relationship between SMRS and probability of death in development set.

**Figure 2:**
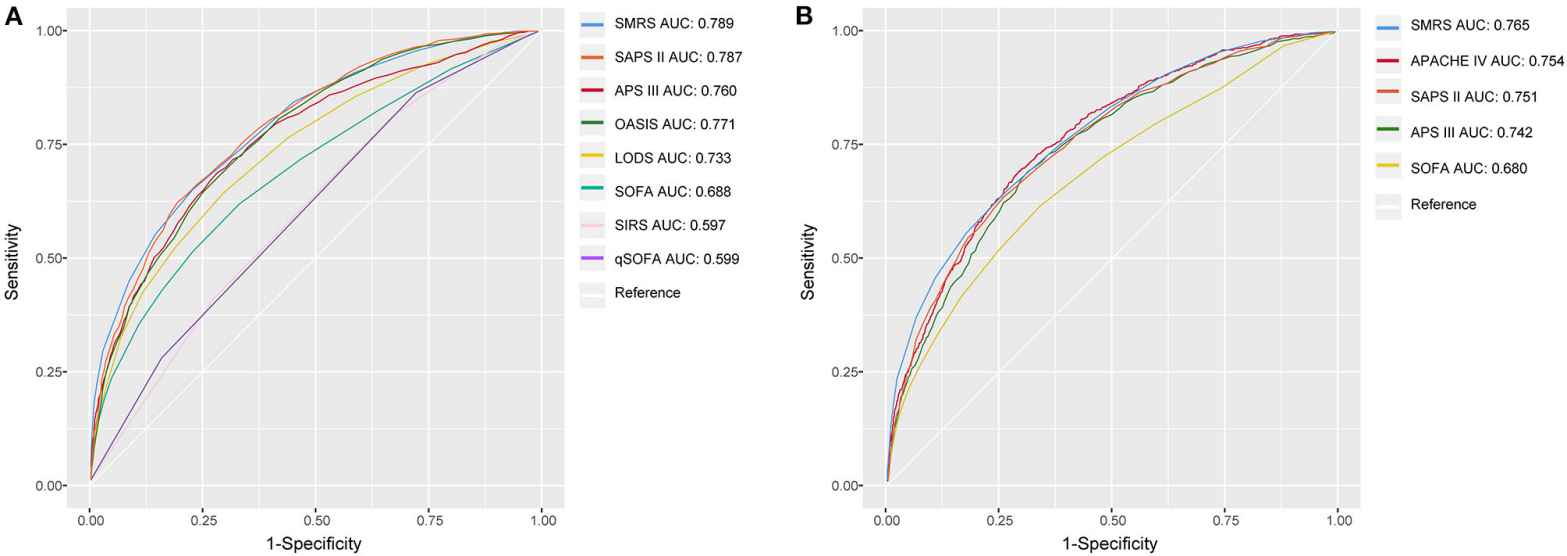
The ROC curves of SMRS and other severity scores. **(A)** Development set; **(B)** Validation set.

**Figure 3:**
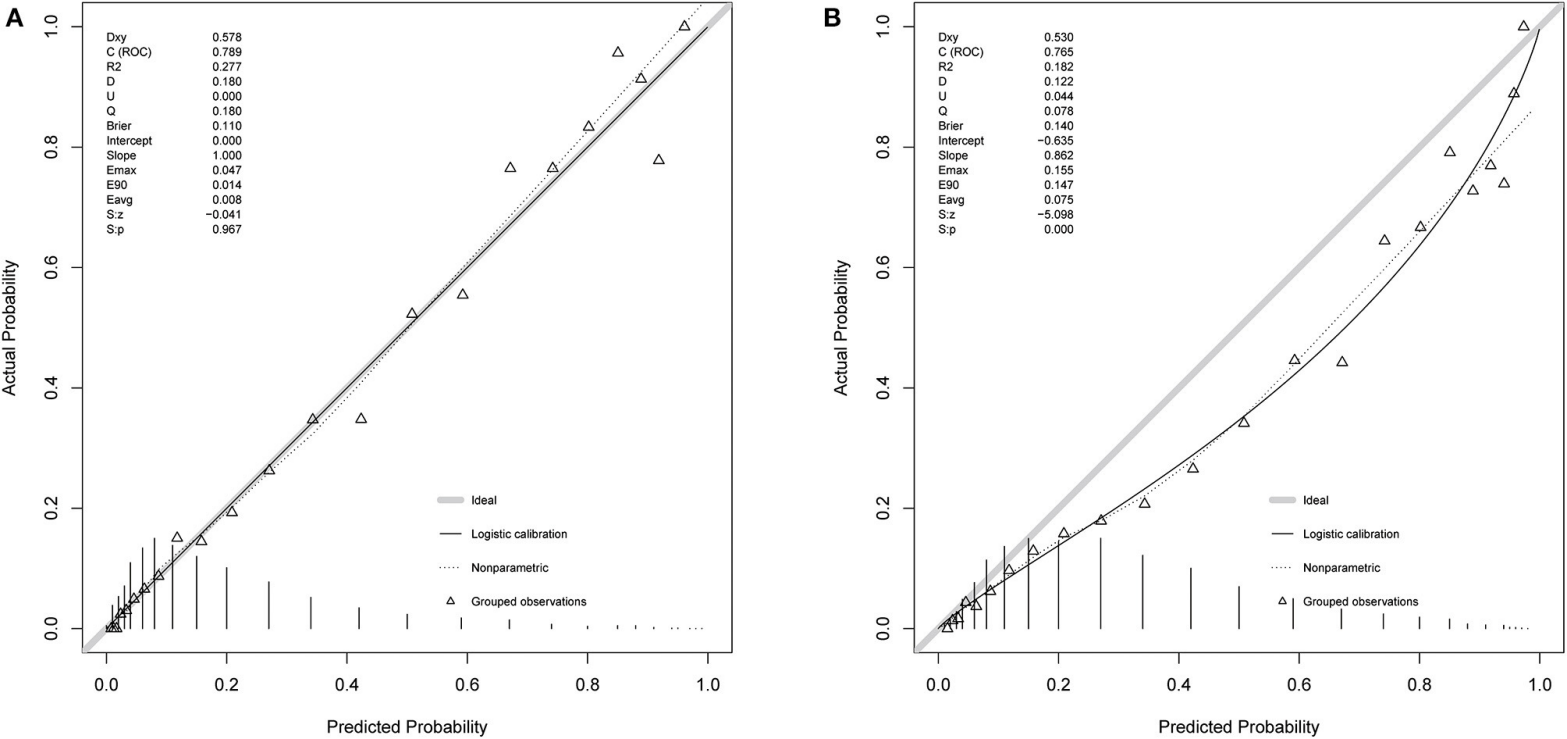
Calibration of SMRS. **(A)** Development set; **(B)** Validation set.

Based on 5,443 patients in the development set in the LASSO model, 35 features were reduced to 15 potential predictors (Additional File 4). After screening, 13 predictors were entered into the LASSO regression model (Additional File 5), and the VIF proved there was no significant multicollinearity in the model (VIF < 5). Additional File 6 shows loess smoothing curves. The SMRS was composed of 13 factors, and the total score range was 0 to 34 (Table 2). The relationship between SMRS and the probability of death is shown in Figure 1, and there was an increasing risk of death with a higher score. The SMRS had good discrimination (AUC: 0.789) in the development set, which was better than other severity scores (Figure 2A). The calibration of SMRS in the development set was shown in Figure 3A. The calibration slope was 1.000 and the Brier value was 0.110. Mortality rates of low (3.2%, 0–6 points), moderate (12.4%, 7–11 points), high (30.7%, 12–14 points), and very high (68.1%, ≥15 points) were yielded by the risk groups for the development set.

**Table 2:** Sepsis mortality risk score.

**Variables**	**Cutoff**	**Score**
Race	Black	0
	White	1
	Others	2
Age (years old)	<45	0
	≥45 and <60	2
	≥60 and <75	3
	≥75	5
Need mechanical ventilation	Yes	2
Lactate (mmol/L)	<4.5	0
	≥4.5 and <8	1
	≥8	3
Temperature (°C)	≥36 and <39	0
	≥39	2
	≥35 and <36	2
	<35	5
SBP (mm/Hg)	>100	0
	≥90 and <100	1
	<90	4
SpO_2_ (%)	≥90	0
	≥80 and <90	1
	<80	2
BUN (mg/dL)	<20	0
	≥20 and <30	1
	≥30	2
WBC (10^9^/L)	≥4 and ≤12	0
	<4	1
	>12 and ≤20	1
	>20	2
Ca (mg/dL)	≥8 and ≤11	0
	≥7 and <8	1
	>11	1
	<7	3
HR (min^−1^)	>100	1
RR (min^−1^)	>22	2
INR	>1.5	1
**Top score**		34

*SBP, Systolic Blood Pressure; SpO_2_, Surplus pulse O_2_; WBC, White Blood cell Count; BUN, Blood Urea Nitrogen; INR, International Normalized Ratio; HR, Heart Rate; RR, Respiratory Rate*.

### Model Performance

**Figure 4:**
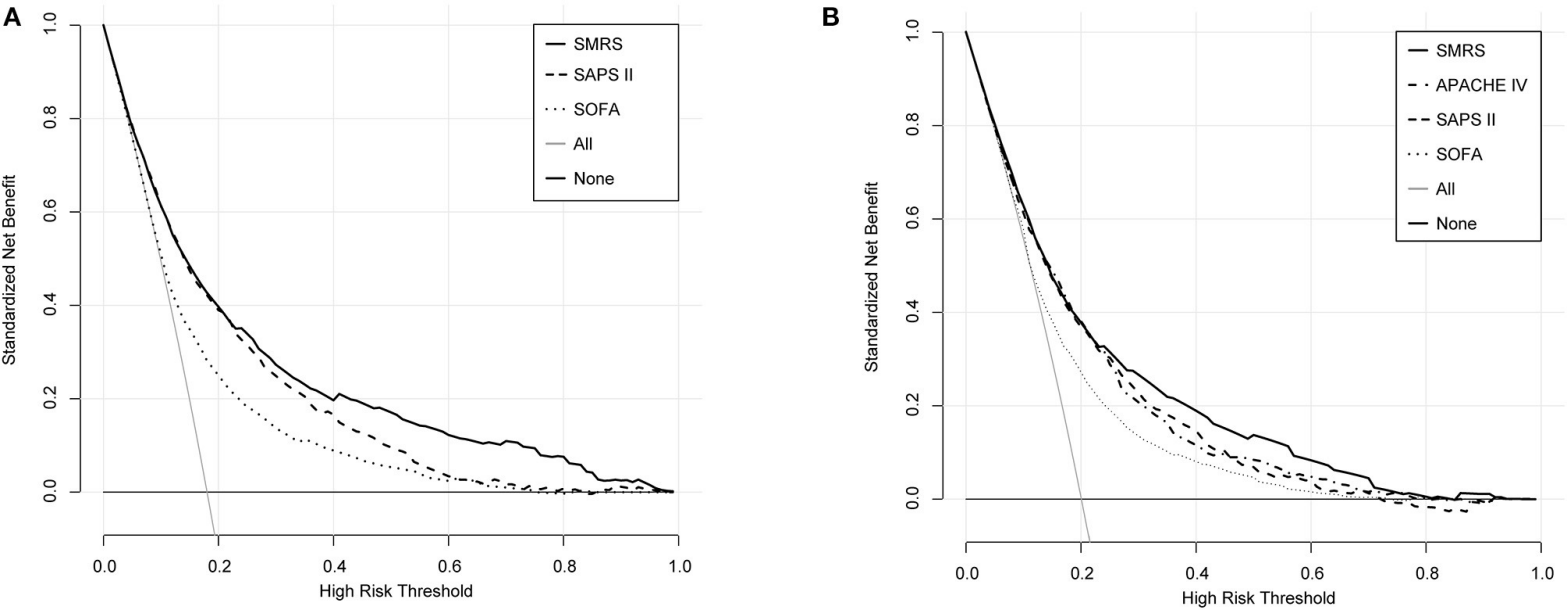
Decision curve analysis of SMRS, SAPS II, SOFA, and APACHE IV. **(A)** Development set; **(B)** Validation set.

In the validation set, we evaluated the discrimination and calibration of SMRS. SMRS was well-discriminated in the external validation set (AUC: 0.765), which was greater than APACHE IV and SAPS II (AUC: APACHE IV 0.754, SAPS II 0.751; Figure 2B). However, no statistical significance of AUCs was observed (De Long method, SMRS vs. APACHE IV: *P*-value 0.221; SMRS vs. SAPS II: *P*-value 0.177). Moreover, the calibration slope was 0.862, and the Brier value was 0.140, indicating that the score has a moderate fit (Figure 3B). For predicting 30-day mortality, the DCA results of SMRS, SAPS II, SOFA, and APACHE IV were shown in Figure 4. A positive net benefit between the threshold probabilities of 10 to 80% was observed through SMRS. The net benefit of SMRS was comparable to SAPS II and APACHE IV and was better than the SOFA in this range.

**Figure 5:**
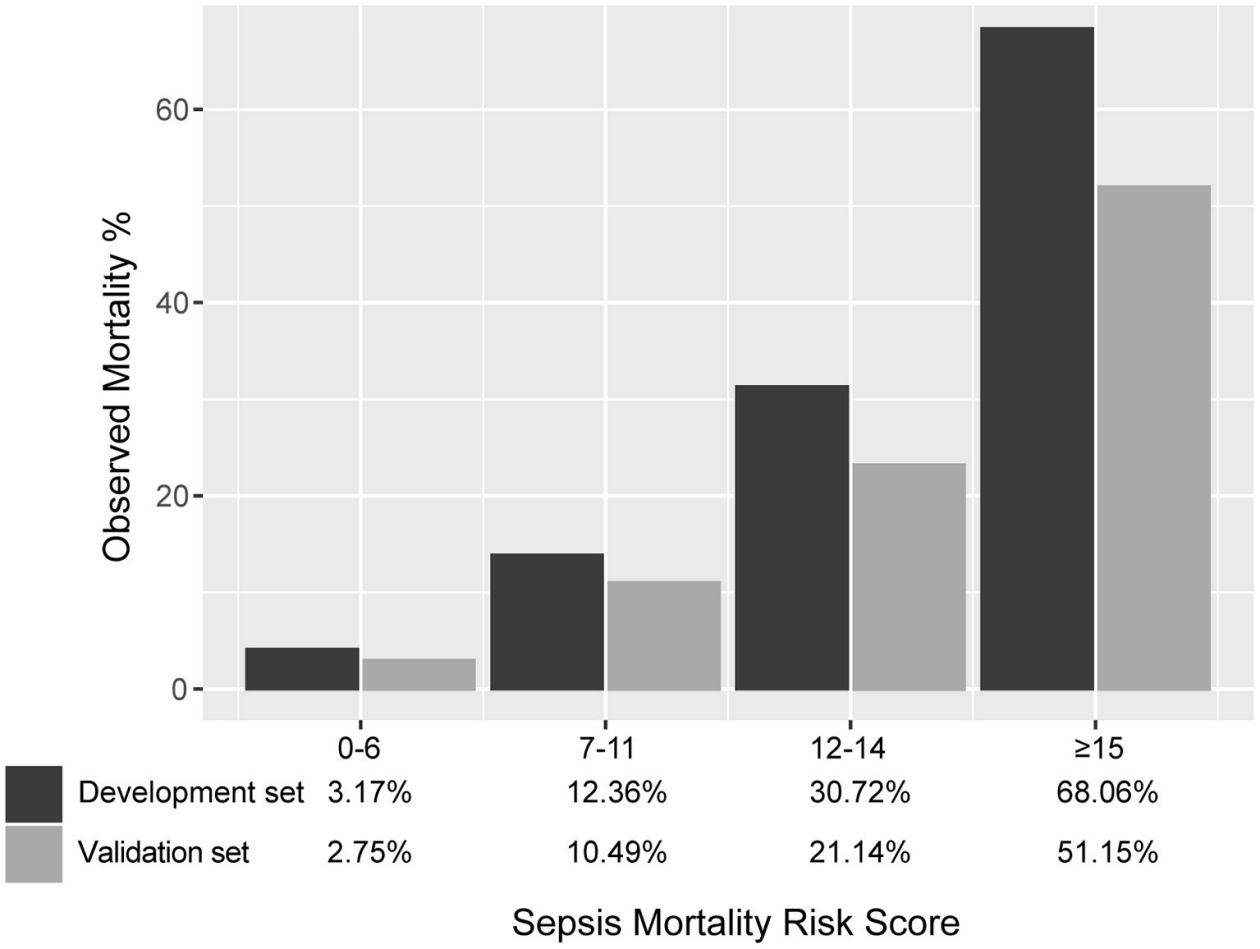
Mortality risk groups according to SMRS.

SMRS accurately stratified patients from the validation set into groups with increased risk of death: low (2.8%), moderate (10.5%), high (21.1%), and very high (51.2%) (Figure 5). The detailed mortality rate stratified by SMRS was reported in Additional File 7.

**Figure 6:**
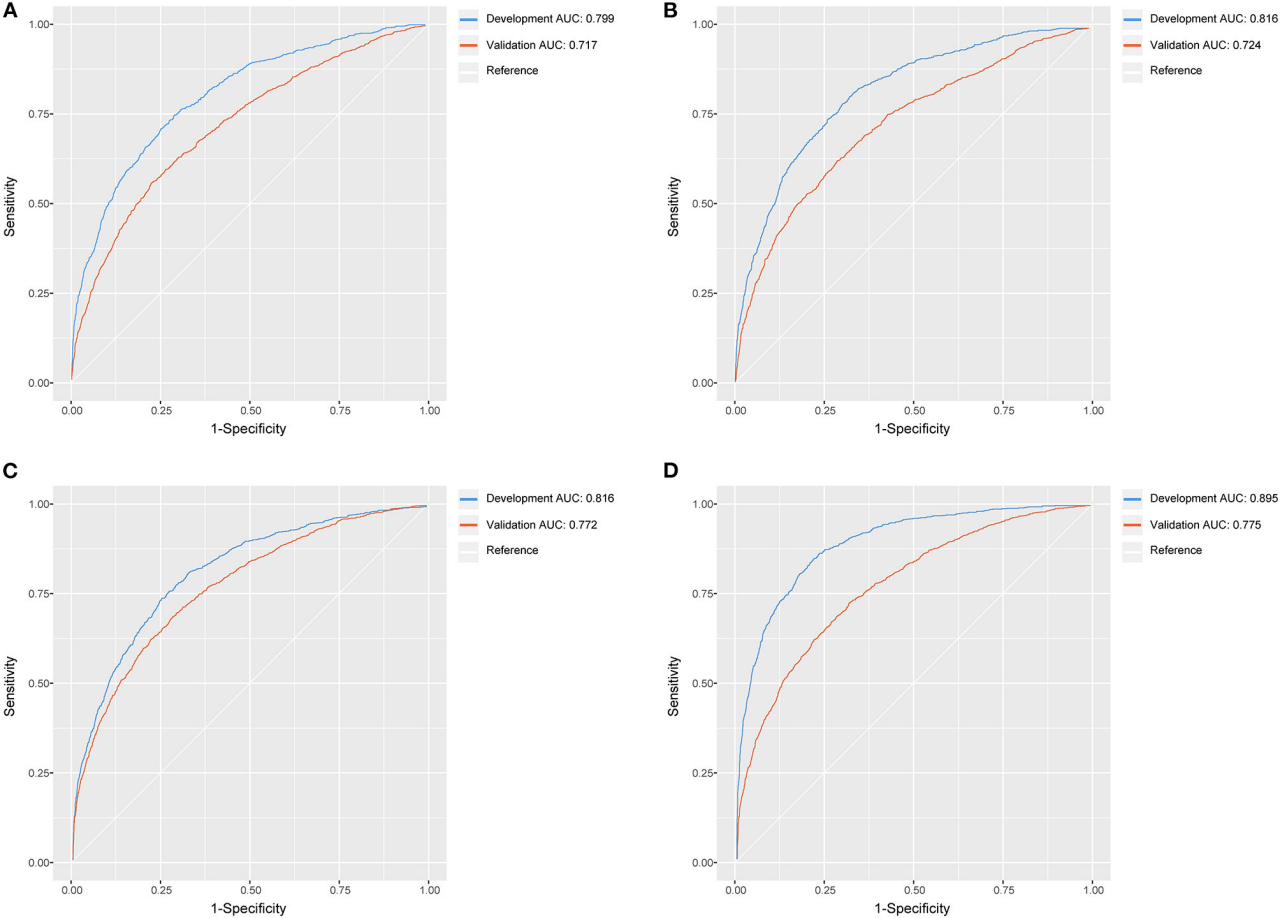
The ROC curves of logistic regression model, MARS model, random forest model, XGBoost model. **(A)** Logistic regression model; **(B)** MARS model; **(C)** random forest model; **(D)** XGBoost model.

All machine learning models, except the logistic regression model, showed good discrimination ability in the development set (AUC > 0.8). In the development and validation sets, the XGBoost algorithm achieved the best performance among the four models (Figure 6).

## Discussion

We used the LASSO method in this study to select the most useful predictive features from the primary sepsis-3 data set, which is suitable for the regression of high-dimensional data (23, 24). Then, we developed a new scoring system, the SMRS. It showed a moderate performance in predicting 30-day mortality and risk-stratifying specifically for ICU patients with sepsis. To identify septic patients, an important strength of our study was the use of new sepsis-3 criteria, and this method would overcome some inherent weaknesses of using hospital discharge data (13, 15). The SMRS contains only 13 simple variables recorded in clinical routines. Therefore, if implemented, the SMRS will not require manual input of additional variables as the model is based on variables routinely collected [the frequently used SAPS II and APACHE IV scores for mortality prediction in the ICU required manually adding additional data (25)]. In the validation set, the discrimination of SMRS was comparable to APACHE IV and SAPS II and was significantly better than the SOFA.

For many years, various scoring systems have been widely used in the ICU, but the ability of general ICU severity scores is insufficient in accurately and reliably predicting mortality in the sepsis patient population. Arabi et al. evaluated four scoring systems in ICU patients with sepsis, reporting poor calibration for all four scores (10). Specifically, the SOFA score was proposed for the sepsis population, and a greater SOFA score was associated with a higher mortality rate (26). However, the SOFA score has several limitations, such as low mortality discrimination power and limited number of variables (27). For predicting mortality in septic patients, the reported AUC of the initial SOFA score ranged from 0.69 to 0.83(28, 29). In our study, for predicting 30-day mortality, the SOFA score had a low discriminatory power (AUC: 0.69). Unlike other ICU severity scores, the SOFA score was developed to describe organ dysfunction and morbidity instead of mortality prediction, and some strong predictors for mortality were not included.

Therefore, specifically for the sepsis-3 population, we aimed at constructing a mortality risk score. For the 35 clinical features, 13 useful predictive features were finally identified using the LASSO method by examining the predictor–outcome association. A two-fold increase in the odds of death was observed in our model in patients requiring mechanical ventilation within the first 24 h of admission. This was because mechanical ventilation among septic patients was typically due to the concomitant acute respiratory distress syndrome, an early sign of poor clinical outcome in sepsis (6). Similarly, many studies have indicated that a strong predictor of mortality for septic patients is serum lactate (30, 31), which, however, was not included in existing risk scores. Since lactate measurement has become a clinical routine, we assigned three or six points to lactate in the final risk score. In our study and previous research, other variables such as hypothermia, hypotension, and advanced age were found to be associated with increased mortality (11, 13, 14, 32, 33).

The SMRS is simple for calculation and easy to use, and has robust discrimination and calibration. When we used SMRS to evaluate patients, DCA results indicated that 80% probability could be considered sufficient to assess mortality risk accurately. To predict the mortality risk of patients with sepsis, ICU physicians could use the SMRS and improve clinical decision-making at the bedside. Moreover, the predictor variables that we used were quite universally obtained in the emergency department (ED). After further validation and recalibration, the SMRS appeared to have the potential to help ED clinicians triage decisions and ICU placement.

In addition, machine learning techniques showed having high potentials to be used in the sepsis population. For predicting mortality among septic patients, the proposed models, particularly the XGBoost model, outperformed traditional scoring systems, including SAPS II and SOFA. However, even though machine learning models offer improved performance for predicting 30-day mortality, practical application in clinical practice has not always been straightforward. Among different populations, the applicability of machine learning models might be limited by heterogeneity (34). An external validation study is required to assess performance and ensure generalizability as the clinical implementation of models is currently scarce. Another major issue in the clinical application is the black-box problem (35, 36). Although these models had high accuracy, their utility has been critically limited due to difficulty in interpretation.

## Limitations

The study has the following limitations. First, we chose to analyze the patients admitted to the ICU with sepsis. There were certainly patients who had been diagnosed with sepsis before or after the ICU admission, but we limited our study population to those who fulfilled sepsis-3 criteria during their first ICU day. Second, we retrospectively identified the septic patient dataset for developing SMRS from a single-center and excluded some patients due to missing data. A few of the variables were also excluded for the same reason, but previous research has shown that they might be associated with septic patients' mortality (e.g., BMI, albumin) (37, 38). Third, in accordance with other severity scores, the timing of variable measurement was determined. If the sampling time was relatively late, the predictive accuracy improved because variables were measured close to the outcome's occurrence, but the timeliness of the prediction was compromised (39). Thus, the use of 24 h after ICU admission was a trade-off between timeliness and prediction accuracy. Furthermore, we conducted an external validation by using the data of 5,658 septic patients from the eICU database, and the results indicated that the calibration of SMRS was relatively poor with an overestimate of 30-day mortality. Finally, we prepared our data set for developing SMRS from 2008 to 2012, and the outcomes of septic patients could have changed over time due to the update of treatment guidelines and advances in treatment and diagnostic technology.

## Conclusion

The probability of septic patients' mortality could accurately be estimated by the SMRS, developed on 5,443 septic patients and validated on 5,658 patients. It is a simple score that can be applied in clinical practice. Therefore, further evaluation regarding its clinical application value is required. In the future, prospective validation and refining of our scoring system across diverse patient populations should be included.

## Data Availability Statement

The raw data supporting the conclusions of this article will be made available by the authors, without undue reservation.

## Author Contributions

KZ and SZ conceived the idea, performed the analysis, and drafted the manuscript. WC and YH interpreted the results and helped to revise the manuscript. GZ and ZZ helped to frame the idea of the study and helped to analyze the data. All authors read and approved the final manuscript.

## Conflict of Interest

The authors declare that the research was conducted in the absence of any commercial or financial relationships that could be construed as a potential conflict of interest.

## Supplementary Material

Additional File 1 TRIPOD checklist.

Additional File 2 Missing values.

Additional File 3 Comparisons of basic characteristics between development and validation sets.

Additional File 4 LASSO and random forest approach.

Additional File 5 Final predictors in the LASSO regression model.

Additional File 6 Loess smoothing curves of continuous variables.

Additional File 7 Mortality rate stratified by SMRS.

## Abbreviations

ICU: Intensive care unit
MIMIC III: Medical Information Mart for Intensive Care III
LASSO: Least Absolute Shrinkage and Selection Operator
SMRS: Sepsis Mortality Risk Score
APACHE II: Acute Physiology and Chronic Health Evaluation II
SAPS II: Simplified Acute Physiology Score II
SOFA: Sequential Organ Failure Assessment
APS III: Acute Physiological Score III
LODS: Logistic Organ Dysfunction System
OASIS: Oxford Acute Severity of Illness Score
SIRS: System Inflammatory Reaction Syndrome
qSOFA: quick SOFA
VIF: variance inflation factor
AUC: Area Under the Curve
DCA: decision curve analysis
MARS: multivariate adaptive regression splines
XGBoost: eXtreme Gradient Boosting
ED: emergency department.
