# Supplementary material for: Development and Validation of a Sepsis Mortality Risk Score for Sepsis-3 Patients in Intensive Care Unit
Source: Front Med (Lausanne). 2021 Jan 21;7:609769. doi: 10.3389/fmed.2020.609769 (PMC7859108; doi:10.3389/fmed.2020.609769)
Supplement: Additional File 2 — Missing values. [file Table_2.DOCX]

Additional File 2 Missing values

|  | N | Missing | |
| --- | --- | --- | --- |
|  |  | Count | Percent |
| Lactate | 4457 | 986 | 18.1 |
| Aniongap | 5418 | 25 | 0.5 |
| Bicarbonate | 5427 | 16 | 0.3 |
| Chloride | 5430 | 13 | 0.2 |
| Hematocrit | 5424 | 19 | 0.3 |
| Hemoglobin | 5421 | 19 | 0.3 |
| Platelet | 5418 | 25 | 0.5 |
| Potassium | 5430 | 13 | 0.2 |
| PTT | 5052 | 391 | 7.2 |
| INR | 5076 | 367 | 6.7 |
| PT | 5076 | 367 | 6.7 |
| Sodium | 5430 | 13 | 0.2 |
| Bun | 5427 | 16 | 0.3 |
| WBC | 5419 | 24 | 0.4 |
| Neutrophil | 4738 | 705 | 13.0 |
| RDW | 5427 | 16 | 0.3 |
| RBC | 5427 | 16 | 0.3 |
| HR | 5441 | 2 | 0.1 |
| SBP | 5432 | 11 | 0.2 |
| DBP | 5432 | 11 | 0.2 |
| MBP | 5441 | 2 | 0.1 |
| RR | 5440 | 3 | 0.1 |
| Temperature | 5332 | 111 | 2.0 |
| SpO2 | 5439 | 4 | 0.1 |
| Glucose | 5392 | 51 | 0.9 |
