# Supplementary material for: Development and Validation of a Sepsis Mortality Risk Score for Sepsis-3 Patients in Intensive Care Unit
Source: Front Med (Lausanne). 2021 Jan 21;7:609769. doi: 10.3389/fmed.2020.609769 (PMC7859108; doi:10.3389/fmed.2020.609769)
Supplement: Additional File 3 — Comparisons of basic characteristics between development and validation sets. [file Table_3.DOCX]

**Additional File 3** Baseline Characteristics of participants between development and validation set

| **Variables** | **Development set (n=5,443)** | **Validation set (n=5,658)** | **P value** |
| --- | --- | --- | --- |
| **Age, years** | 67.0 (54.0-80.0) | 67.0 (56.0-78.0) | 0.549 |
| **Gender, n** |  |  | 0.008 |
| Male | 3,020 (55.5) | 3,011 (53.2) |  |
| Female | 2,423 (44.5) | 2,647 (46.8) |  |
| **Ethnicity, n** |  |  | <0.001 |
| White | 3,945 (72.5) | 4,464 (78.9) |  |
| Black | 475 (8.7) | 456 (8.1) |  |
| Others | 1,023 (18.8) | 738 (13.0) |  |
| **Admission type, n** |  |  | <0.001 |
| Emergency | 5,061 (93.0) | 2,732 (48.3) |  |
| Others | 382 (7.0) | 2,926 (51.7) |  |
| **Comorbidities, n** |  |  |  |
| Heart failure | 957 (17.6) | 843 (14.9) | 0.001 |
| Hypertension | 868 (15.9) | 731 (12.9) | <0.001 |
| COPD | 1,103 (20.3) | 1,093 (19.6) | 0.167 |
| Diabetes | 1,563 (28.7) | 1,371 (24.2) | <0.001 |
| Renal failure | 1,000 (18.4) | 942 (16.6) | 0.048 |
| Hepatopathy | 544 (10.0) | 179 (3.2) | <0.001 |
| Lymphoma | 95 (1.7) | 80 (1.4) | 0.272 |
| **Need mechanical  ventilation, n** | 2,638 (48.5) | 1,934 (34.2) | <0.001 |
| **30-day mortality, n** | 907 (16.7) | 1,135 (20.1) | <0.001 |

Data are expressed as frequencies (percentage) or median (interquartile range). The results of the comparison between the two groups was analyzed by Mann-Whitney test for continuous variables or the chi-squared test for categorical variables.

RRT: Renal Replacement Therapies; COPD: Chronic Obstructive Pulmonary Disease.
