# Supplementary material for: Development and Validation of a Sepsis Mortality Risk Score for Sepsis-3 Patients in Intensive Care Unit
Source: Front Med (Lausanne). 2021 Jan 21;7:609769. doi: 10.3389/fmed.2020.609769 (PMC7859108; doi:10.3389/fmed.2020.609769)

**Additional File 4** LASSO and random forest approach


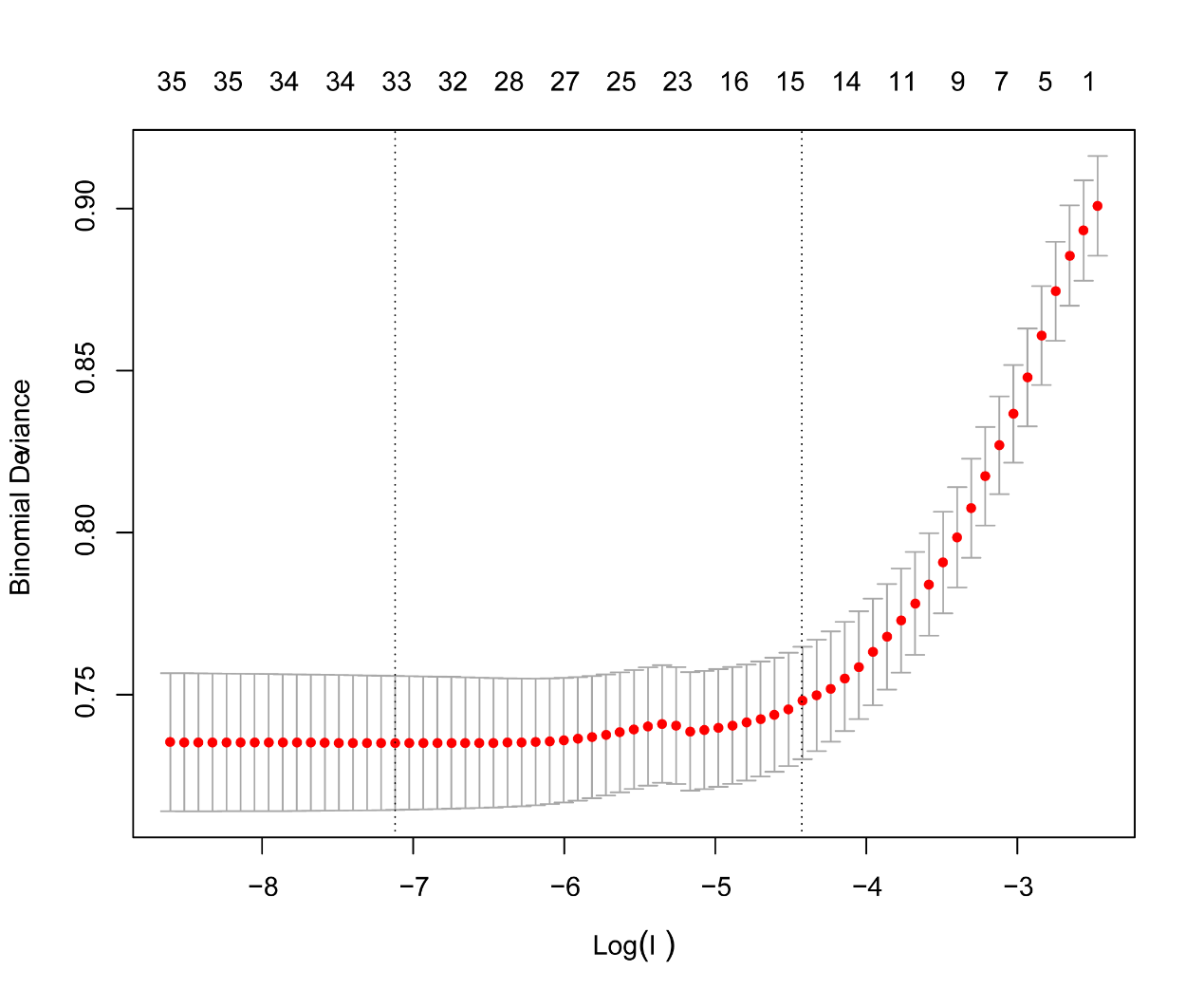


| Variables | Coefficients |
| --- | --- |
| Age | 0.025298756 |
| Race | 0.203621372 |
| Admission | 0.431454289 |
| MV | 0.597435543 |
| HR | 0.002948059 |
| SBP | -0.007207658 |
| RR | 0.077281605 |
| Temperature | -0.272988442 |
| SpO2 | -0.020704644 |
| Lactate | 0.086805579 |
| Aniongap | 0.028664597 |
| INR | 0.018805008 |
| BUN | 0.005878192 |
| WBC | 0.006753541 |
| Ca | -0.012523415 |


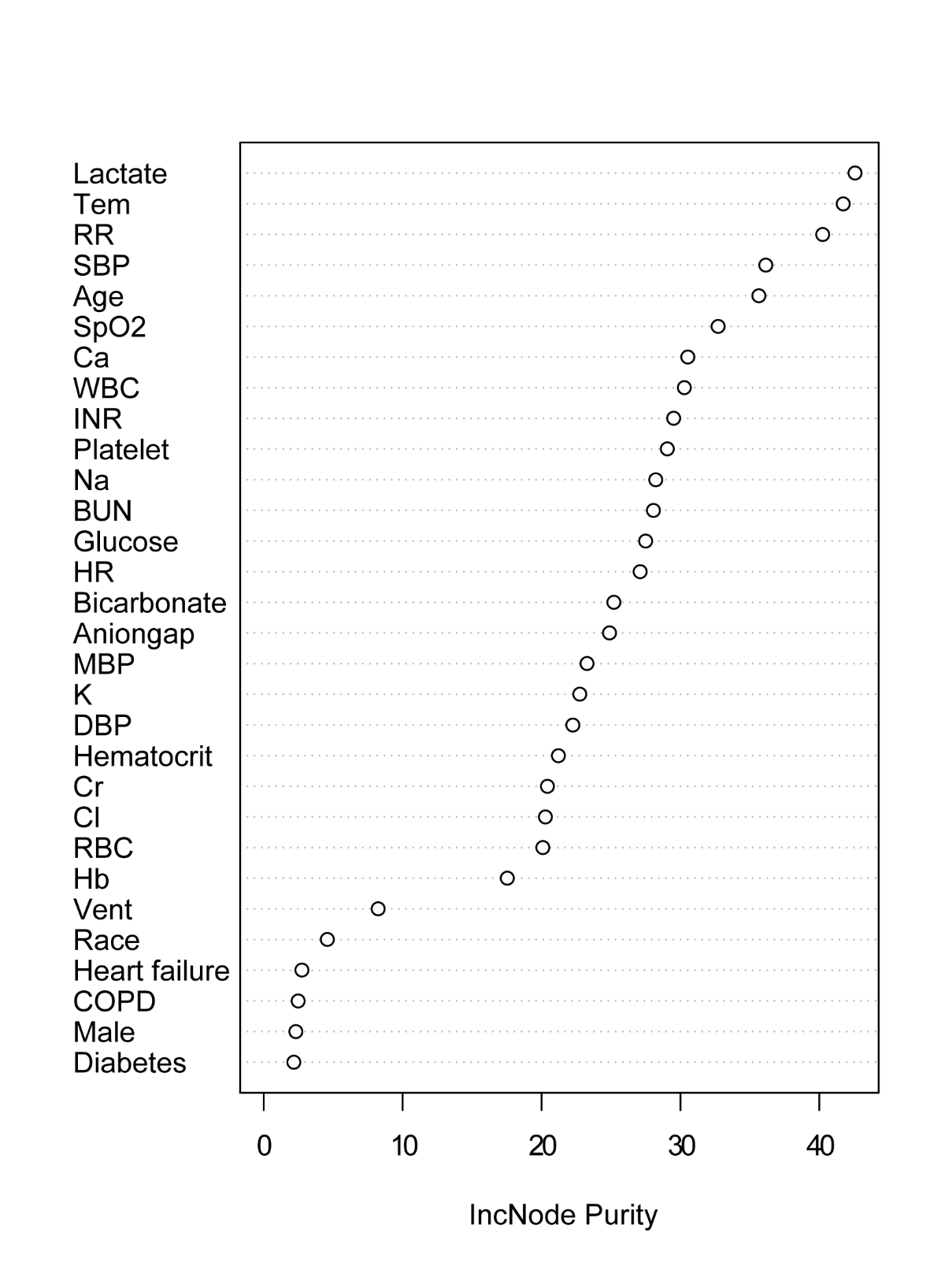

Supplement: Additional File 4 — LASSO and random forest approach. [file Table_4.DOCX]
