# Supplementary material for: Development and Validation of a Sepsis Mortality Risk Score for Sepsis-3 Patients in Intensive Care Unit
Source: Front Med (Lausanne). 2021 Jan 21;7:609769. doi: 10.3389/fmed.2020.609769 (PMC7859108; doi:10.3389/fmed.2020.609769)
Supplement: Additional File 5 — Final predictors in the LASSO regression model. [file Table_5.DOCX]

**Additional File 6** Final predictors in the LASSO regression model

| Variables | Coefficient | P value | Exp(B) | 95% CI for EXP(B) | |
| --- | --- | --- | --- | --- | --- |
|  |  |  |  | Lower | Upper |
| Need mechanical ventilation | 0.773 | <0.001 | 2.166 | 1.825 | 2.572 |
| Age |  |  |  |  |  |
| <45 | Reference |  |  |  |  |
| ≥45 and <60 | 0.608 | 0.001 | 1.836 | 1.270 | 2.654 |
| ≥60 and <75 | 0.937 | <0.001 | 2.553 | 1.789 | 3.643 |
| ≥75 | 1.580 | <0.001 | 4.854 | 3.424 | 6.880 |
| HR≥100 | 0.204 | 0.014 | 1.227 | 1.043 | 1.442 |
| SBP |  |  |  |  |  |
| >100 | Reference |  |  |  |  |
| ≥90 and <100 | 0.377 | 0.003 | 1.459 | 1.138 | 1.870 |
| <90 | 1.310 | <0.001 | 3.708 | 2.332 | 5.895 |
| RR | 0.596 | <0.001 | 1.814 | 1.523 | 2.161 |
| Temperature |  |  |  |  |  |
| ≥36 and <39 | Reference |  |  |  |  |
| ≥39 or ≥35 and <36 | 0.748 | <0.001 | 2.113 | 1.624 | 2.749 |
| <35 | 1.528 | <0.001 | 4.607 | 2.433 | 8.723 |
| SpO_2_ |  |  |  |  |  |
| ≥90 | Reference |  |  |  |  |
| ≥80 and <90 | 0.312 | 0.002 | 1.367 | 1.117 | 1.672 |
| <80 | 0.682 | <0.001 | 1.978 | 1.434 | 2.729 |
| Lactate |  |  |  |  |  |
| <4.5 | Reference |  |  |  |  |
| ≥4.5 and <8 | 0.284 | 0.018 | 1.328 | 1.049 | 1.681 |
| ≥8 | 1.146 | <0.001 | 3.146 | 2.26 | 4.381 |
| INR | 0.329 | <0.001 | 1.39 | 1.173 | 1.647 |
| BUN |  |  |  |  |  |
| <20 | Reference |  |  |  |  |
| ≥20 and <30 | 0.367 | 0.002 | 1.443 | 1.145 | 1.818 |
| ≥30 | 0.685 | <0.001 | 1.984 | 1.621 | 2.428 |
| WBC |  |  |  |  |  |
| ≥4 and ≤12 | Reference |  |  |  |  |
| <4 or >12 and ≤20 | 0.387 | <0.001 | 1.473 | 1.236 | 1.756 |
| >20 | 0.727 | <0.001 | 2.07 | 1.622 | 2.641 |
| Calcium |  |  |  |  |  |
| ≥8 and ≤11 | Reference |  |  |  |  |
| ≥7 and <8 or >11 | 0.296 | 0.003 | 1.344 | 1.122 | 1.611 |
| <7 | 1.114 | <0.001 | 3.046 | 2.059 | 4.505 |
| Race |  |  |  |  |  |
| Black | Reference |  |  |  |  |
| White | 0.376 | 0.026 | 1.456 | 1.046 | 2.028 |
| Others | 0.718 | <0.001 | 2.05 | 1.426 | 2.948 |

HR: Heart Rate; RR: Respiratory Rate; SBP: Systolic Blood Pressure; SpO_2_: Surplus pulse O_2_; BUN: Blood Urea Nitrogen; INR: International Normalized Ratio.

We excluded three predictors for the following reasons:

1. Emergency admission: the proportion of emergency admission was too high, more than 90% of participants got points and the predictive ability was limited;
2. Aniongap: there is potential interactive relationship between aniongap and lactate.
