# Supplementary figures and images for: Development and Validation of a Sepsis Mortality Risk Score for Sepsis-3 Patients in Intensive Care Unit
Source: Front Med (Lausanne). 2021 Jan 21;7:609769. doi: 10.3389/fmed.2020.609769 (PMC7859108; doi:10.3389/fmed.2020.609769)

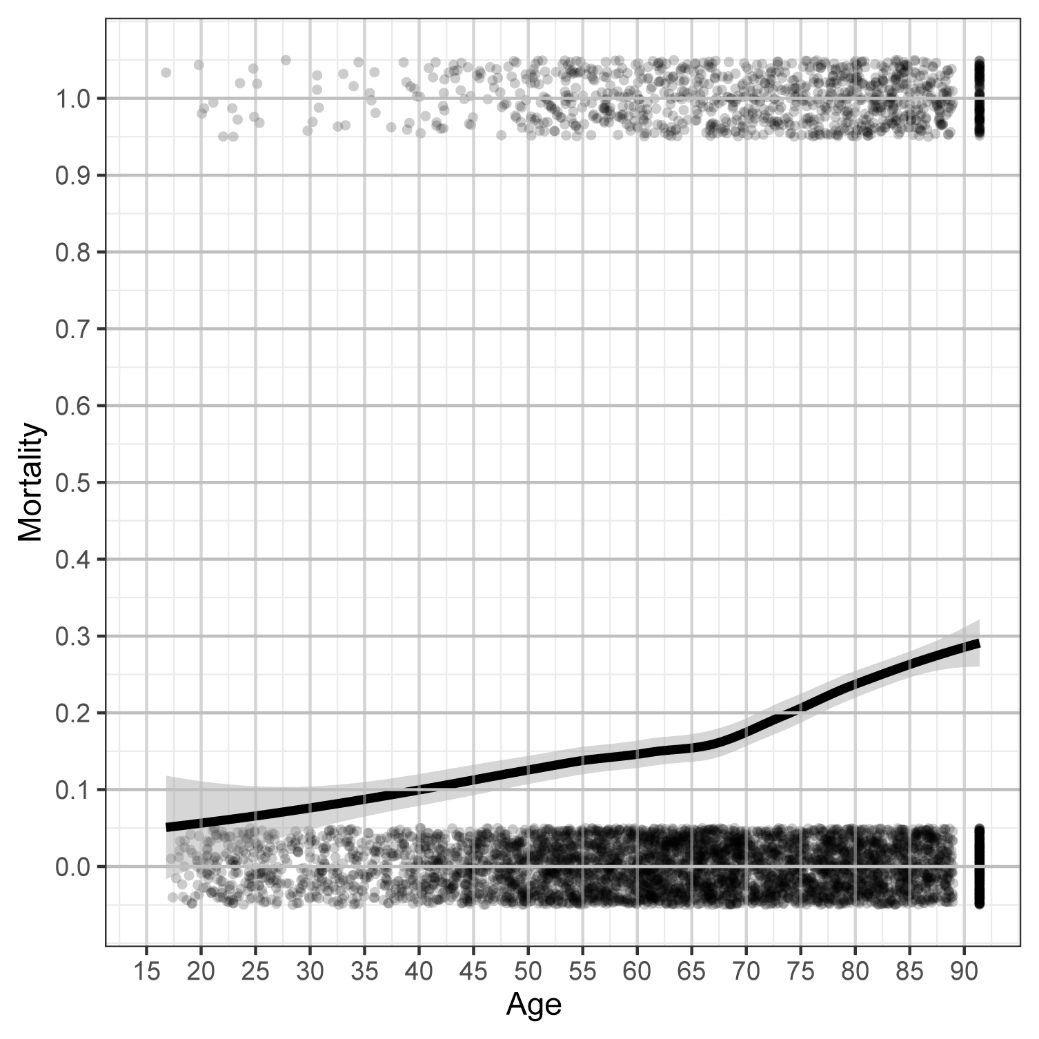

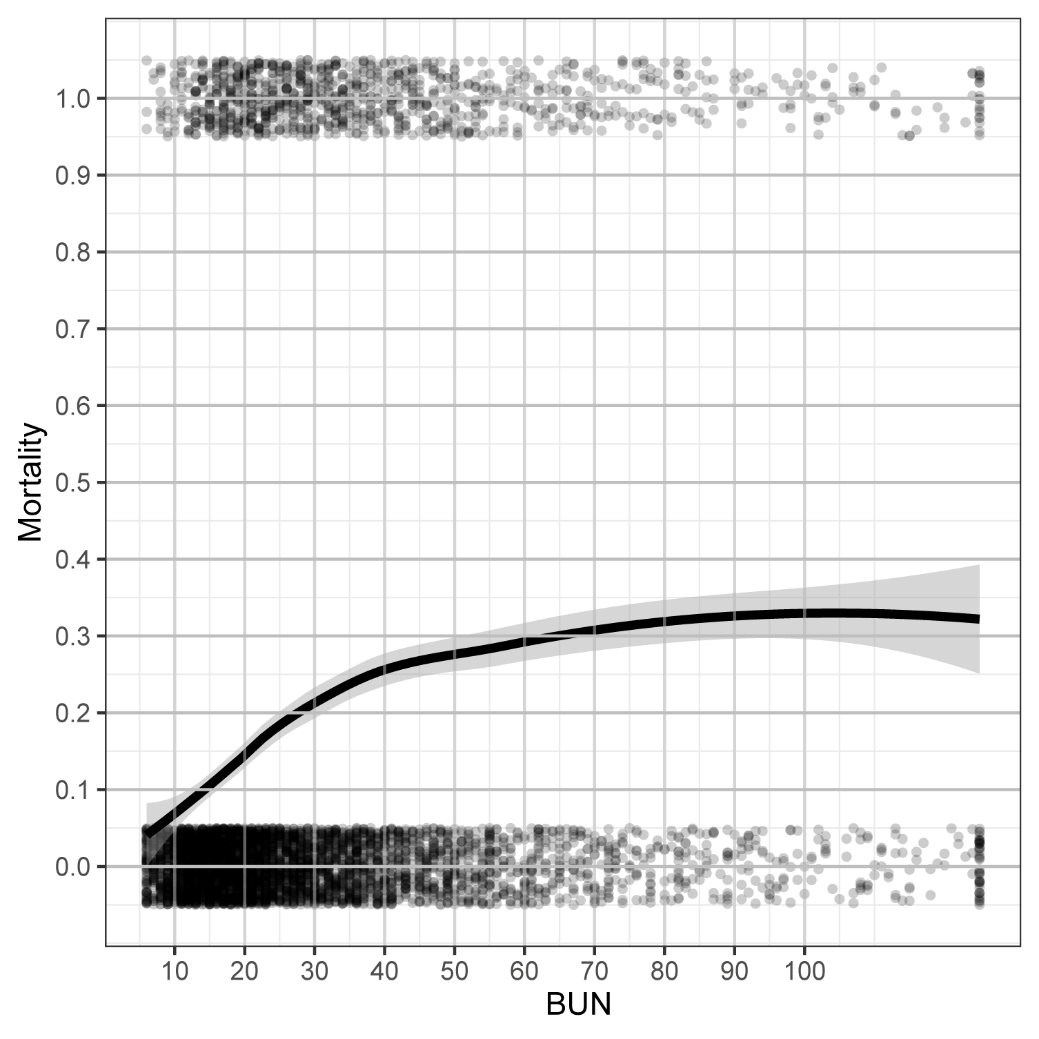

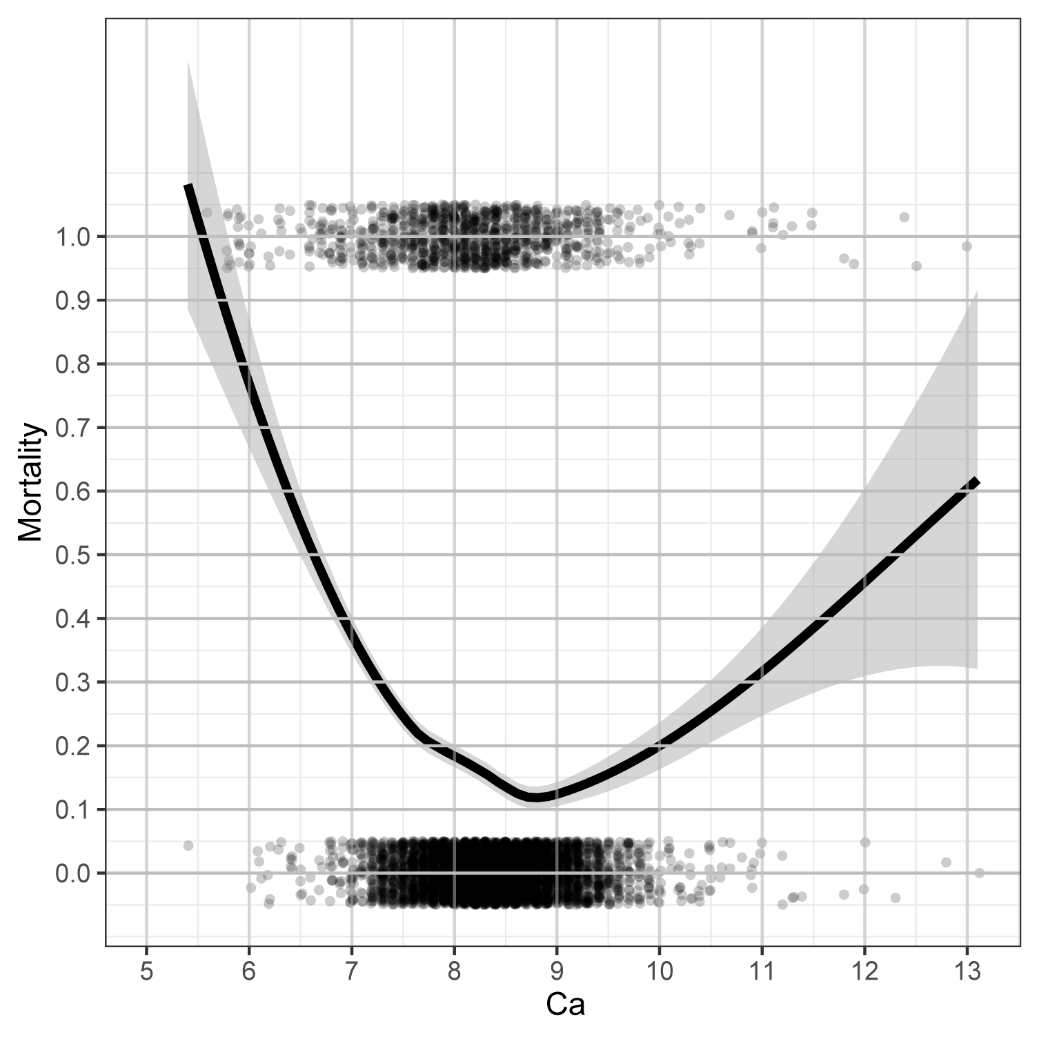

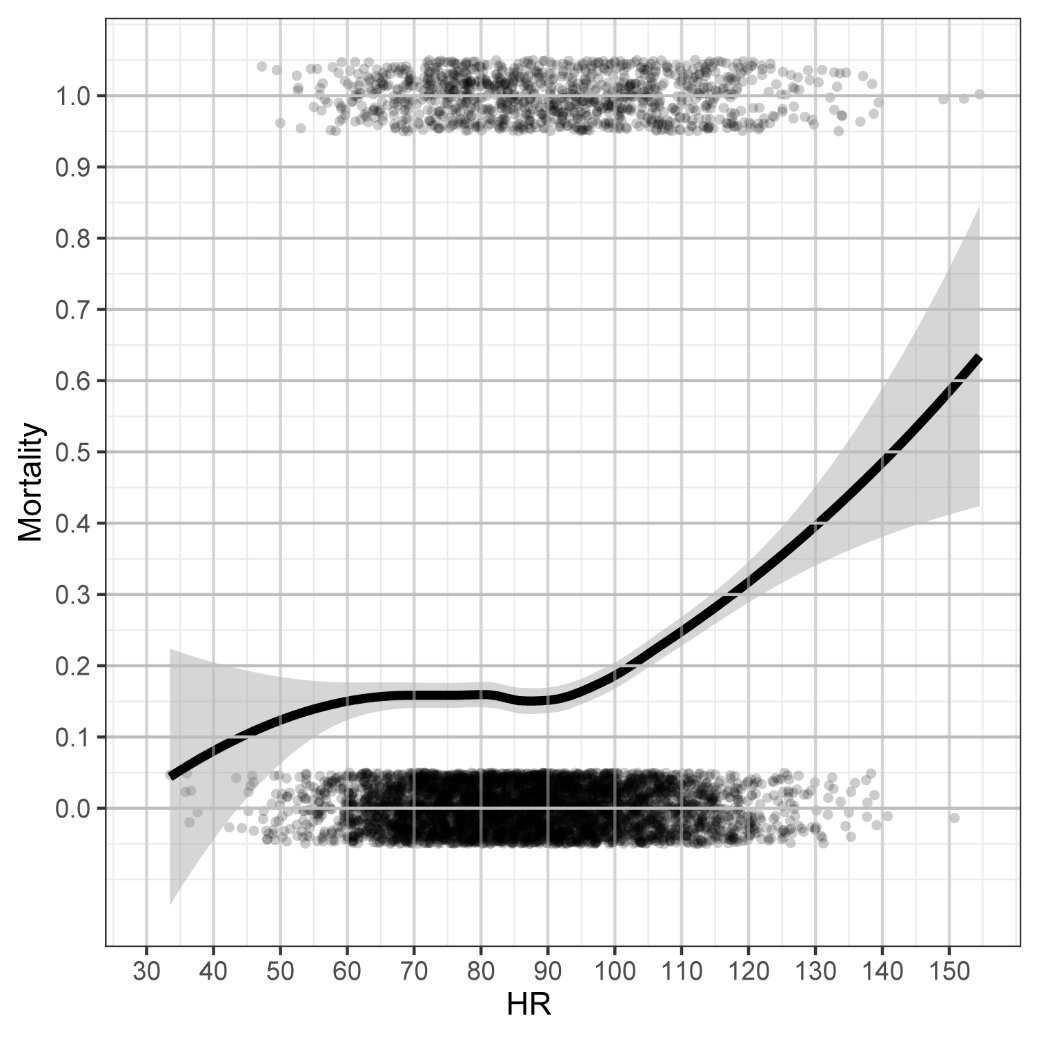

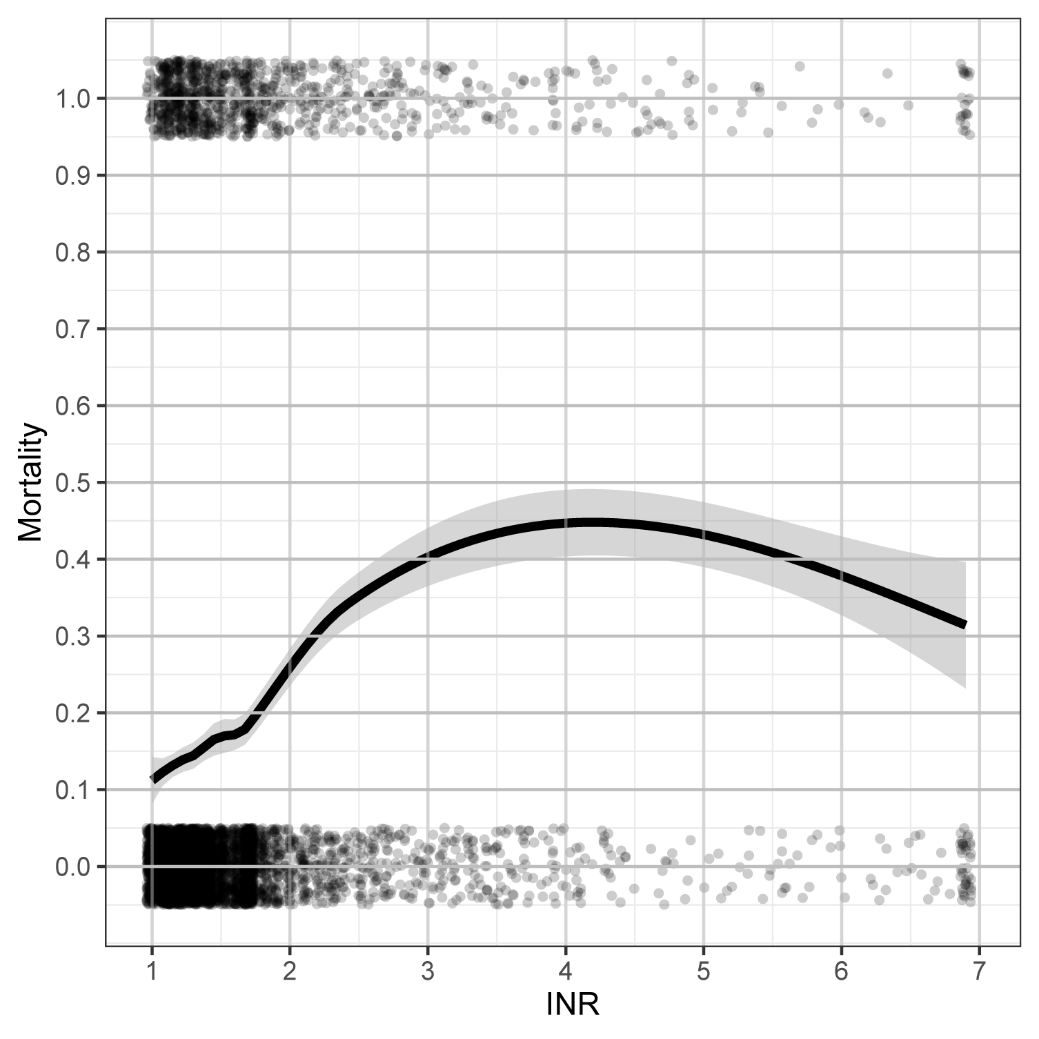

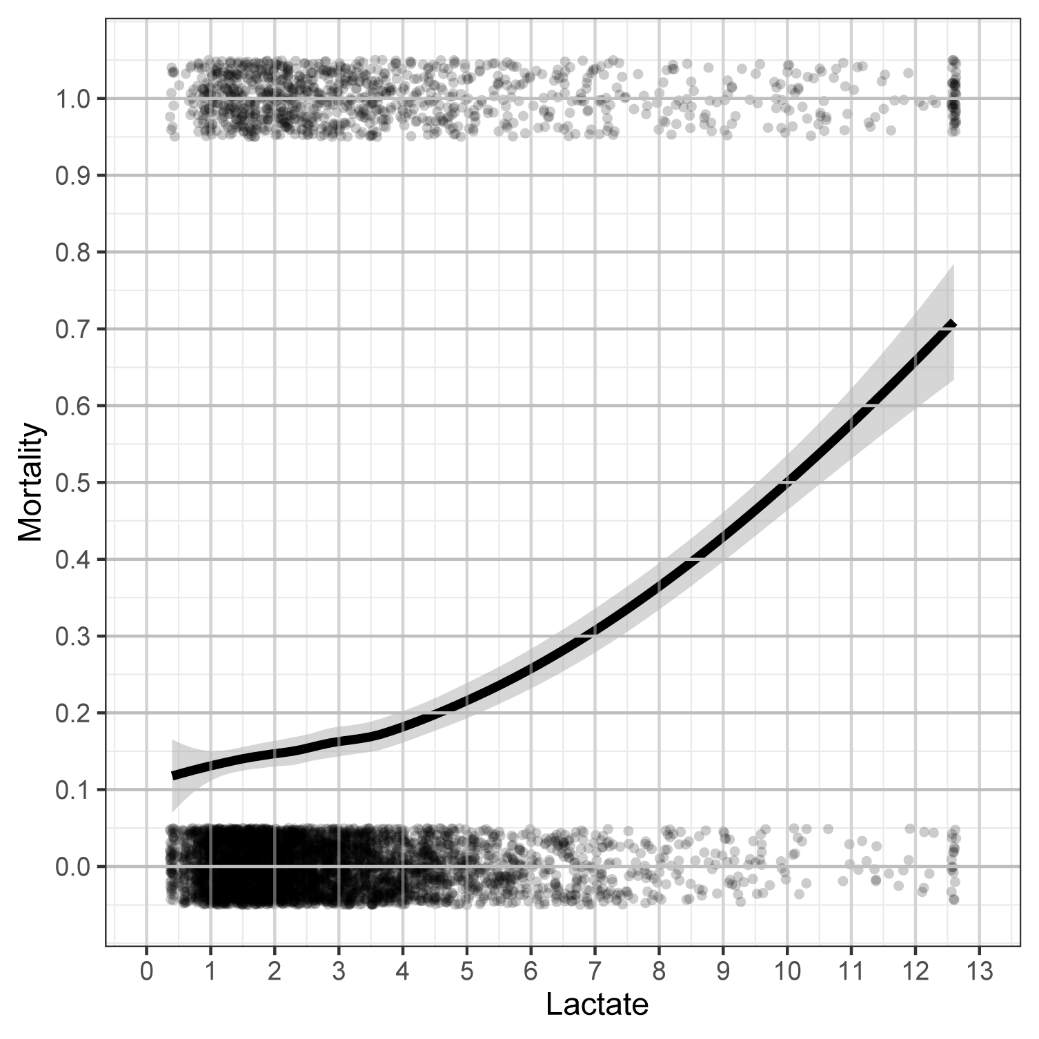

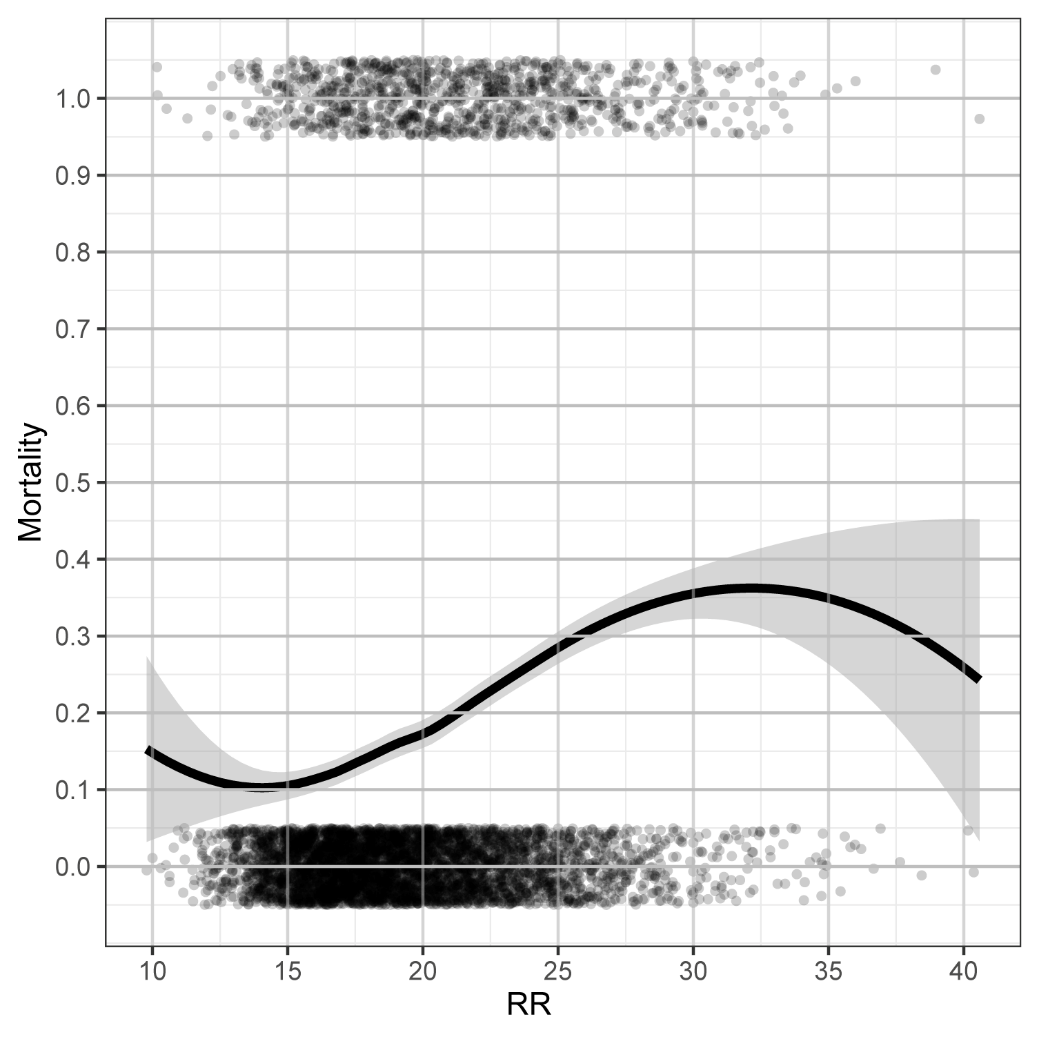

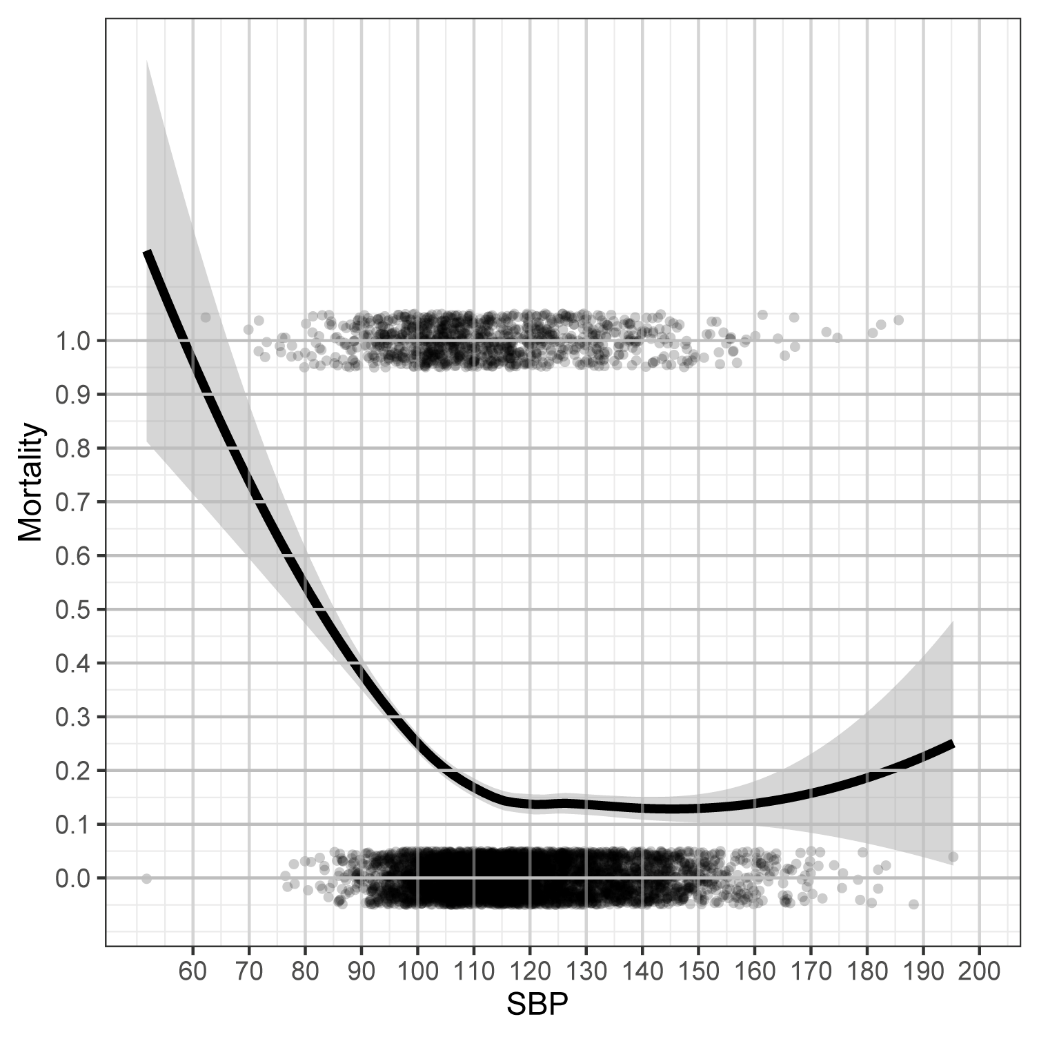

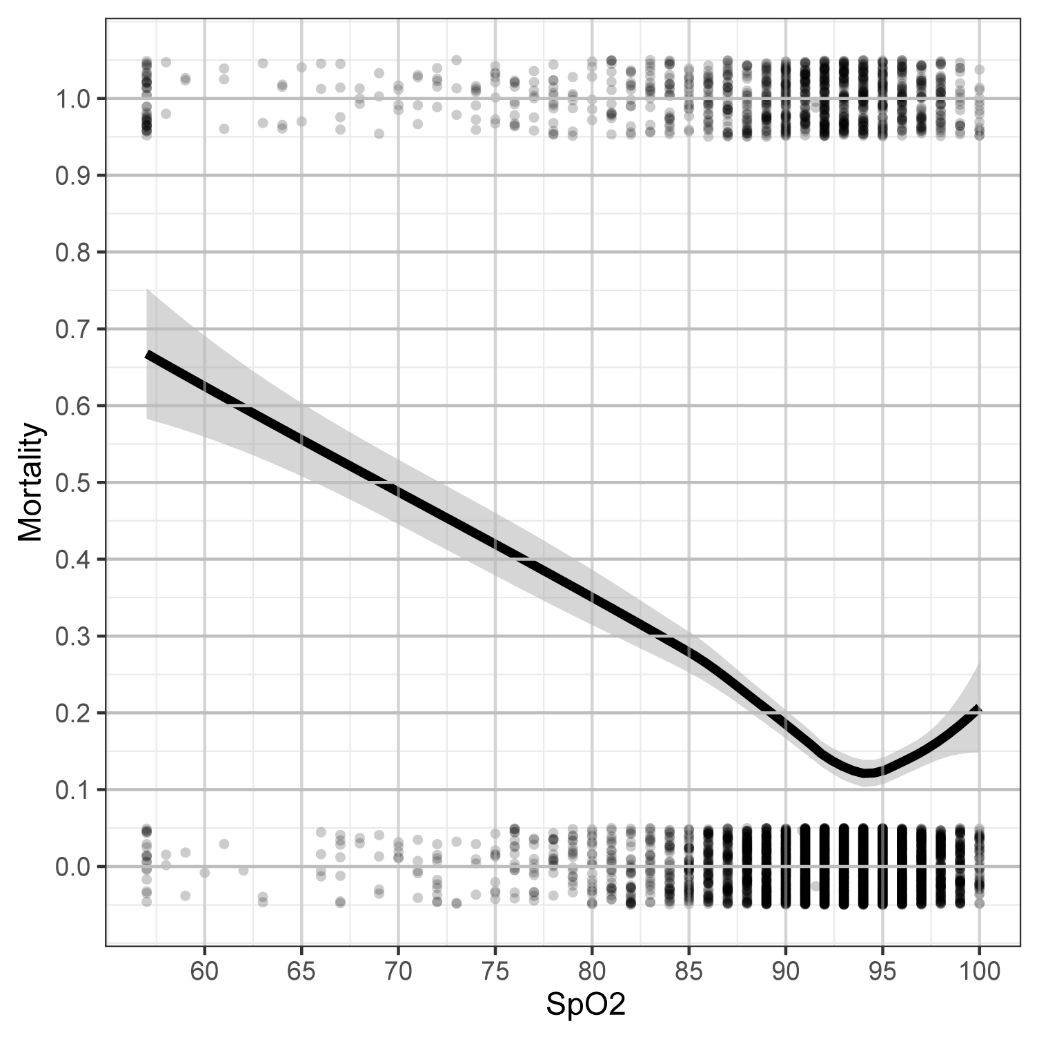

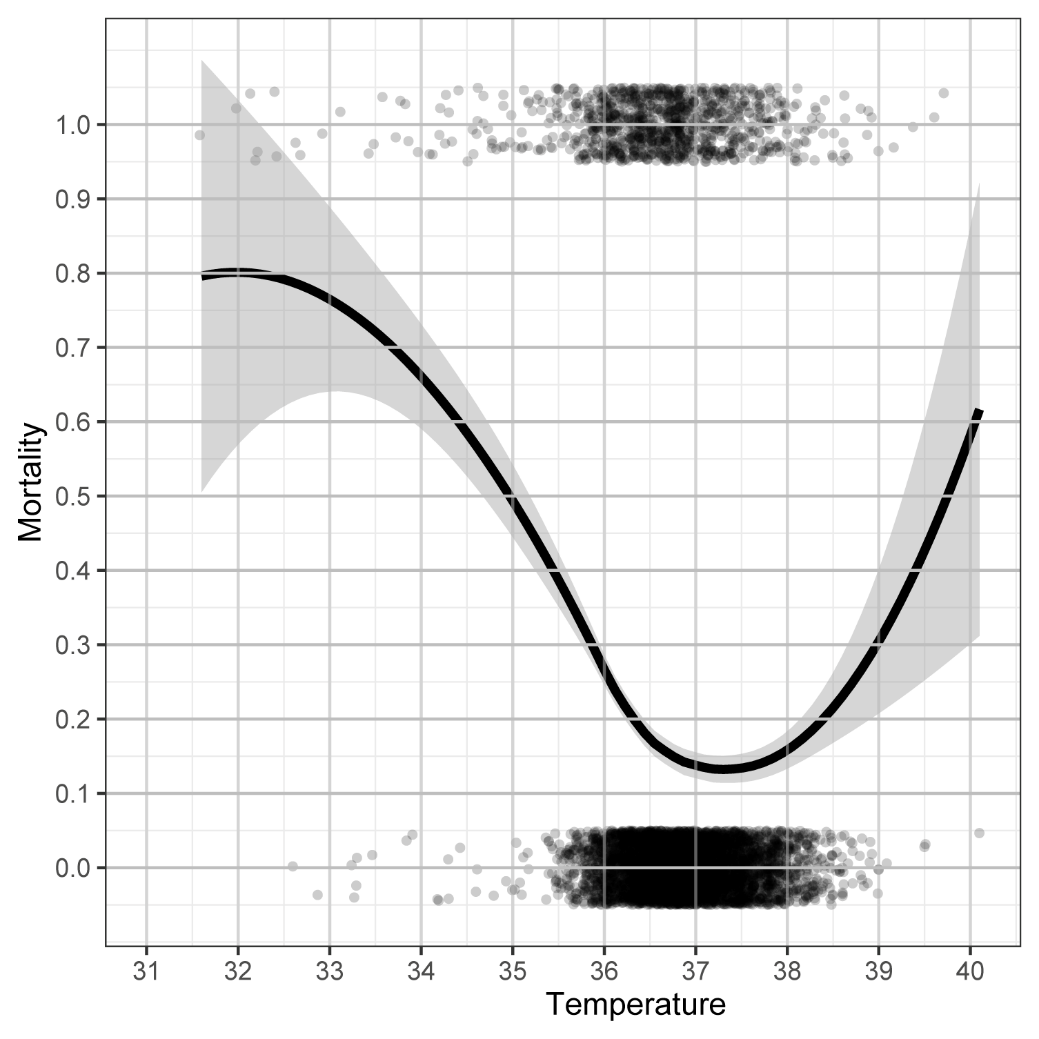

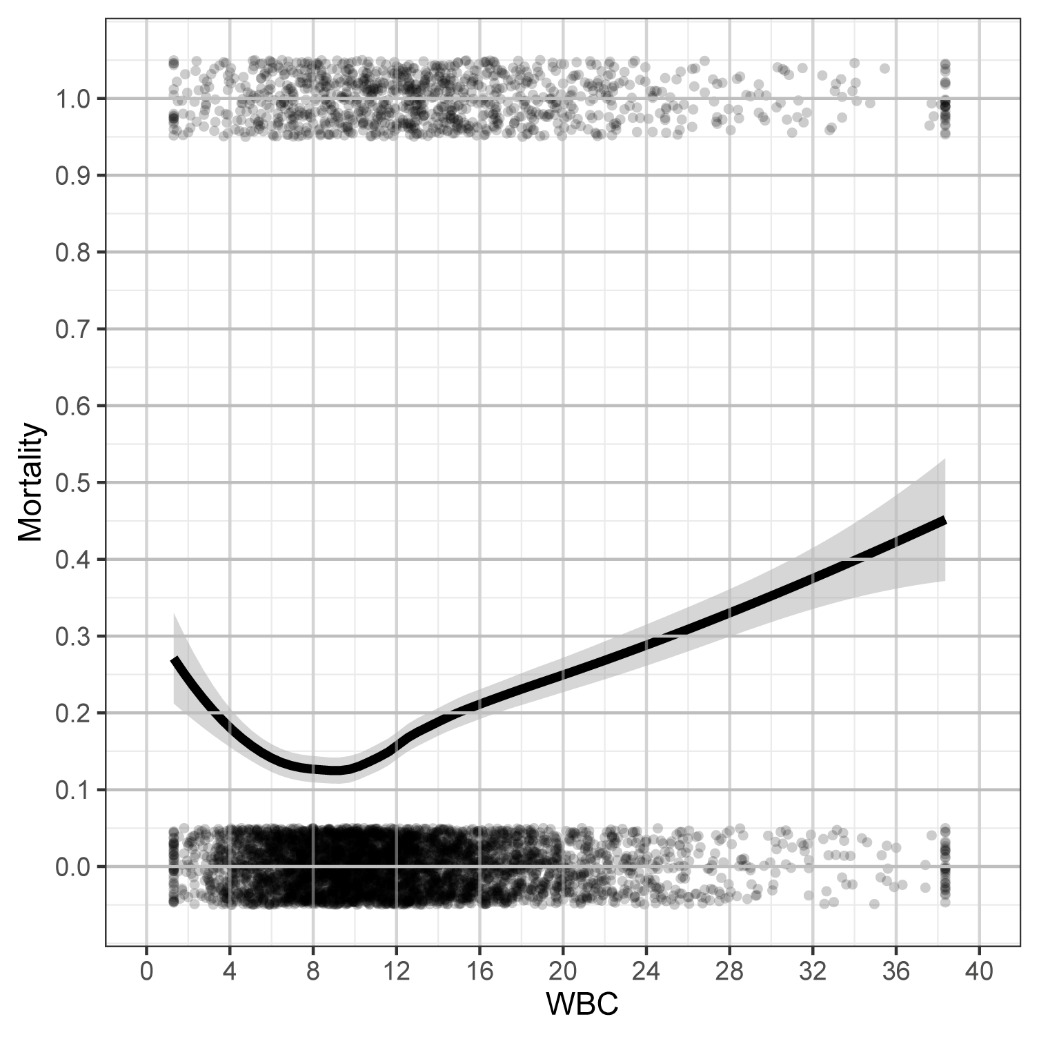

Supplement: Additional File 6 — Loess smoothing curves of continuous variables. [file Table_6.DOCX]
