# Supplementary material for: Development and Validation of a Sepsis Mortality Risk Score for Sepsis-3 Patients in Intensive Care Unit
Source: Front Med (Lausanne). 2021 Jan 21;7:609769. doi: 10.3389/fmed.2020.609769 (PMC7859108; doi:10.3389/fmed.2020.609769)
Supplement: Additional File 7 — Mortality rate stratified by SMRS. [file Table_7.DOCX]

**Additional File 7** Mortality rate according to Sepsis Mortality Risk Score group

| Score | Survivors | Non-survivors | Total | Mortality% |
| --- | --- | --- | --- | --- |
| Development set | |  |  |  |
| 0-6 | 1253 | 41 | 1294 | 3.17 |
| 7-11 | 2631 | 371 | 3002 | 12.36 |
| 12-14 | 530 | 235 | 765 | 30.72 |
| ≥15 | 122 | 260 | 382 | 68.06 |
| Total | 4536 | 907 | 5443 | 16.66 |
|  | |  |  |  |
| Validation set | |  |  |  |
| 0-6 | 425 | 12 | 437 | 2.75 |
| 7-11 | 2363 | 277 | 2640 | 10.49 |
| 12-14 | 1246 | 334 | 1580 | 21.14 |
| ≥15 | 489 | 512 | 1001 | 51.15 |
| Total | 4523 | 1135 | 5658 | 20.06 |

Development set

| Score | Survivors | Non-survivors | | All | | Mortality% | | |
| --- | --- | --- | --- | --- | --- | --- | --- | --- |
| 0 | 5 | 0 | | 5 | | 0.00 | | |
| 1 | 18 | 0 | | 18 | | 0.00 | | |
| 2 | 58 | 0 | | 58 | | 0.00 | | |
| 3 | 122 | 0 | | 122 | | 0.00 | | |
| 4 | 243 | 6 | | 249 | | 2.41 | | |
| 5 | 321 | 10 | | 331 | | 3.02 | | |
| 6 | 486 | 25 | | 511 | | 4.89 | | |
| 7 | 584 | 41 | | 625 | | 6.56 | | |
| 8 | 639 | 61 | | 700 | | 8.71 | | |
| 9 | 548 | 97 | | 645 | | 15.04 | | |
| 10 | 479 | 81 | | 560 | | 14.46 | | |
| 11 | 381 | 91 | | 472 | | 19.28 | | |
| 12 | 267 | 95 | | 362 | | 26.24 | | |
| 13 | 158 | 84 | | 242 | | 34.71 | | |
| 14 | 105 | 56 | | 161 | | 34.78 | | |
| 15 | 53 | 58 | | 111 | | 52.25 | | |
| 16 | 37 | 46 | | 83 | | 55.42 | | |
| 17 | 16 | 52 | | 68 | | 76.47 | | |
| 18 | 8 | 26 | | 34 | | 76.47 | | |
| 19 | 3 | 15 | | 18 | | 83.33 | | |
| 20 | 1 | 22 | | 23 | | 95.65 | | |
| 21 | 2 | 21 | | 23 | | 91.30 | | |
| 22 | 2 | 7 | | 9 | | 77.78 | | |
| 23 | 0 | 4 | | 4 | | 100.00 | | |
| 24 | 0 | 5 | | 5 | | 100.00 | | |
| 26 | 0 | 1 | | 1 | | 100.00 | | |
| 27 | 0 | 1 | | 1 | | 100.00 | | |
| 29 | 0 | 2 | | 2 | | 100.00 | | |
| Total | 4536 | 907 | | 5443 | | 16.66 | | |
| Validation set | | |  | |  | | |  |
| Score | Survivors | Non-survivors | | All | | | Mortality% | |
| 0 | 1 | 0 | | 1 | | | 0.00 | |
| 1 | 1 | 0 | | 1 | | | 0.00 | |
| 2 | 9 | 0 | | 9 | | | 0.00 | |
| 3 | 25 | 0 | | 25 | | | 0.00 | |
| 4 | 73 | 1 | | 74 | | | 1.35 | |
| 5 | 118 | 2 | | 120 | | | 1.67 | |
| 6 | 198 | 9 | | 207 | | | 4.35 | |
| 7 | 313 | 12 | | 325 | | | 3.69 | |
| 8 | 454 | 30 | | 484 | | | 6.20 | |
| 9 | 524 | 56 | | 580 | | | 9.66 | |
| 10 | 554 | 82 | | 636 | | | 12.89 | |
| 11 | 518 | 97 | | 615 | | | 15.77 | |
| 12 | 523 | 114 | | 637 | | | 17.90 | |
| 13 | 410 | 107 | | 517 | | | 20.70 | |
| 14 | 313 | 113 | | 426 | | | 26.53 | |
| 15 | 195 | 101 | | 296 | | | 34.12 | |
| 16 | 117 | 94 | | 211 | | | 44.55 | |
| 17 | 77 | 61 | | 138 | | | 44.20 | |
| 18 | 37 | 67 | | 104 | | | 64.42 | |
| 19 | 27 | 54 | | 81 | | | 66.67 | |
| 20 | 14 | 53 | | 67 | | | 79.10 | |
| 21 | 9 | 24 | | 33 | | | 72.73 | |
| 22 | 6 | 20 | | 26 | | | 76.92 | |
| 23 | 6 | 17 | | 23 | | | 73.91 | |
| 24 | 1 | 8 | | 9 | | | 88.89 | |
| 25 | 0 | 8 | | 8 | | | 100.00 | |
| 26 | 0 | 4 | | 4 | | | 100.00 | |
| 28 | 0 | 1 | | 1 | | | 100.00 | |
| Total | 4523 | 1135 | | 5658 | | | 20.06 | |
